# In Vivo Imaging of the Innate Immune System in the Pancreas in New‐Onset and Long‐Standing Type 1 Diabetes

**DOI:** 10.1111/dom.70672

**Published:** 2026-03-23

**Authors:** Ebrahim Anvari, Hongchao Zhang, Henrik Hill, Nathalia Guarienti Missima, Per Liss, Irina Velikyan, Gunnar Antoni, Olle Korsgren, Daniel Espes, Olof Eriksson

**Affiliations:** ^1^ Science for Life Laboratory Uppsala University Uppsala Sweden; ^2^ Department of Medical Sciences Uppsala University Uppsala Sweden; ^3^ Department of Medicinal Chemistry Uppsala University Uppsala Sweden; ^4^ Department of Women's and Children's Health Uppsala University Uppsala Sweden; ^5^ Department of Surgical Sciences Uppsala University Uppsala Sweden; ^6^ PET Center, Akademiska Sjukhuset Uppsala Sweden; ^7^ Department of Immunology, Genetics and Pathology Uppsala University Uppsala Sweden; ^8^ Department of Medical Cell Biology Uppsala University Uppsala Sweden

**Keywords:** imaging, immune infiltration, innate immune system, innate immunity, positron emission tomography, type 1 diabetes

## Abstract

**Objective:**

Type 1 diabetes (T1D) is an autoimmune disease, but knowledge of immune cell infiltration in the human pancreas is limited due to the risks associated with pancreatic biopsies. This study aimed to evaluate the feasibility of imaging innate immune cells in vivo using positron emission tomography (PET).

**Methods:**

We used PET tracers targeting M1 macrophages ([^68^Ga]‐DOTATATE) and neutrophil elastase ([^11^C]‐NES) to assess pancreatic immune cell infiltration in individuals with new‐onset T1D (*n* = 4), long‐standing T1D (*n* = 6), and healthy controls (*n* = 6).

**Results:**

Both tracers enabled visualisation and quantification of pancreatic uptake. Uptake of both [^68^Ga]‐DOTATATE and [11C]‐NES was not increased in new‐onset T1D compared to healthy controls but was elevated in individuals with long‐standing T1D.

**Discussion:**

PET imaging of innate immune cells in the pancreas is feasible. While no increased macrophage or neutrophil activity was observed in new‐onset T1D, increased tracer uptake in long‐standing T1D may reflect late‐stage inflammatory processes and fibrosis.

## 1 Introduction

Immune‐mediated islet inflammation, that is, insulitis, is a key pathophysiological aspect of type 1 diabetes (T1D). In animal models of T1D, insulitis is apparent and has been extensively studied, while in humans, its origin and spatiotemporal dynamics remain unclear due to the lack of accurate non‐invasive assays. This is largely due to the risks associated with taking pancreatic biopsies and hence most of our knowledge is based on autopsy material or biopsies from brain‐dead organ donors. Recent advancements in Positron Emission Tomography (PET) tracers offer a unique opportunity to non‐invasively study the activity of specific immune cells directly within the pancreas, including macrophages and neutrophils. Macrophages and neutrophils are two important immune cells that have major roles in the innate immune system. Macrophages can have either a pro‐inflammatory (M1) or anti‐inflammatory (M2) phenotype [1]. Interestingly, macrophages are present in islets, where they impact endocrine cells [2, 3, 4]. The Somatostatin Receptor Subtype 2 (SSTR2) is expressed on M1 macrophages but not on other immune cells [5]. The PET tracer [^68^Ga]‐DOTATATE, a selective SSTR2 agonist, is an approved diagnostic agent for localising neuroendocrine tumours (NETs) [6, 7, 8]. Recently, [^68^Ga]‐DOTATATE has been used in humans to visualise M1 macrophages in atherosclerotic plaques, myocarditis and in the lungs [5, 9, 10]. The neutrophil is another innate immune cell that is recruited in the early immune response to combat microorganisms by releasing proteases, such as neutrophil elastase (NE). Neutrophils can also initiate and exacerbate autoimmune disorders, including vasculitis, multiple sclerosis, and systemic lupus erythematosus [11, 12, 13] and have also been found to play a role in the destruction of pancreatic beta‐cells [14]. Increasing evidence indicates that innate immune activation in T1D involves coordinated interactions between neutrophils and macrophages rather than isolated cell populations. Pancreatic macrophages and β‐cells can produce chemokines that recruit neutrophils to the pancreas, whereas activated neutrophils release proteases and NET‐associated mediators that further amplify local inflammation and promote recruitment of monocytes/macrophages. Thus, neutrophil‐mediated tissue injury and macrophage‐associated inflammation may represent temporally related components of the same innate immune cascade [15]. However, the spatiotemporal relationship between these cell populations in the human pancreas remains unknown due to the lack of non‐invasive in vivo methods. The newly developed PET‐tracer [^11^C]GW457427 (abbreviated as [^11^C]‐NES) shows promise in quantifying NE to assess immune‐mediated inflammatory processes in pre‐clinical models including acute respiratory distress syndrome [16] and sepsis [17]. [^11^C]‐NES has recently been clinically evaluated for detection of NE and neutrophils in several indications including COVID‐19 [18, 19] and interstitial lung disease [19]. Furthermore, clinical studies are ongoing in inflammatory bowel disease, lung cancer and rheumatoid arthritis and renal injury (NCT06372444).

In the current study, we aimed to investigate the temporal and spatial distribution of M1 macrophage and neutrophil activity in the pancreas during T1D development by utilising [^68^Ga]‐DOTATATE and [^11^C]‐NES in individuals with new‐onset and long‐standing T1D compared to healthy controls (HC).

## 2 Research Design and Methods

### 2.1 Ethical Statement and Patient Inclusion

The study was approved by the Swedish Ethical Review Authority (approval number 2021–01032) and was conducted in consistency with The Declaration of Helsinki. Inclusion criteria for the T1D groups required confirmed T1D autoantibody positivity. Participants with new‐onset T1D (onset within 3 months) were aged 18–36 years, whereas those with long‐standing T1D (≥ 10 years of disease duration) were aged 18–70 years. Healthy controls were autoantibody negative and aged 18–36 years. Patients with new‐onset T1D were examined with MMTT and PET/MRI within 3 months from diagnosis (range 8–10 weeks since diagnosis) and underwent a second examination 6 months following the first one. HC and patients with long‐standing T1D were examined once.

### 2.2 Metabolic Profiling and Beta‐Cell Function

Clinical examinations, collection of fasting blood samples and assessment of beta‐cell function by a mixed meal tolerance test (MMTT) were conducted within 4 weeks of PET/MRI imaging. The MMTT was conducted at bed rest after overnight fasting. Following oral administration of 360 mL Resource protein (Nestlé Health Science, Switzerland) consumed within 5 min, glucose, pro‐insulin, insulin and C‐peptide were sampled after 15, 30, 60, 90, and 120 min. From the MMTT an area under the curve (AUC) for C‐peptide was computed using the trapezoid formula.

### 2.3 [ ^68^Ga]‐DOTATATE and [ ^11^C]‐NES Radiosynthesis

Both [^68^Ga]‐DOTATATE and [^11^C]‐NES were synthesized according to Good Manufacturing Practice (GMP) as previously described [6, 20].

### 2.4 PET/MRI Examinations

Prior to the PET/MRI examinations participants were fasting for more than 4 h. Before each imaging session, all female participants in fertile age performed a pregnancy test. The participant was placed in supine position in an integrated Signa PET/3 T MRI (GE Healthcare) scanner allowing for simultaneous dynamic PET scanning and MRI sequences. The pancreas was positioned in the center of the PET axial field of view (25 cm) by a scout MRI scan. After positioning, a 60‐min dynamic PET acquisition was performed following intravenous administration of [^11^C]‐NES (target dose of 5 MBq/kg, 353 ± 57 MBq). A minimum interval of 2 h after injection passed to allow for decay of all remaining Carbon‐11 activity. Next, a second 60‐min dynamic scan was acquired using [^68^Ga] DOTATATE (target dose of 3 MBq/kg, 228 ± 34 MBq). For both tracers, image acquisition commenced simultaneously with tracer injection. Anatomical MR images were acquired simultaneously as PET scanning using a 3D LAVA‐Flex (Dixon) gradient echo with water‐only reconstruction for attenuation correction and anatomical reference. Due to a technical failure during the [^68^Ga]‐DOTATATE synthesis one of the new‐onset patients only underwent a [^11^C]‐NES examination when re‐examined after 6 months. PET/MRI images were pseudonymised and transferred to Uppsala University Research Picture Archiving and Communication System (PACS).

MRI images were examined, using the Vue PACS (Philips), in order to exclude clinical pathologies.

### 2.5 PET Data Analysis

The PMOD software (PMOD Technologies, Zürich, Switzerland) was used for PET/MRI image analysis. Pancreatic volume was segmented on trans‐axial projections of T1 MRI images and reported in millilitres (mL). Summation PET images were generated in order to verify that the segmentation was correctly co‐registered. If not, minor manual corrections were made. PET images were analysed using Carimas software (2.10, Turku PET Centre, Finland). Standardised Uptake Values (SUV) were used to quantify tracer uptake, with all measurements based on mean SUV (SUV_mean_). Pancreas segmentations were manually delineated on transaxial planes using the trace tool, taking special care to avoid adjacent high‐uptake organs such as the small intestine and spleen to minimise spill‐in effects. For [^68^Ga]‐DOTATATE, the head/caput was avoided due to the presence of PP‐cells that have expression of SSTRs also at normal physiology. For reference organs (liver, spleen, muscle, and aorta), segmentations were placed on selected planes and interpolated to generate volumes of interest (VOIs). Bone marrow uptake was assessed by placing 1–2 segmentations per vertebral body across multiple planes.

### 2.6 Statistics

Statistical analyses were performed using GraphPad Prism 10.2 (GraphPad Software, Boston, MA, USA). Differences between the groups were computed based on one‐way ANOVA using either Dunnett's or Tukey's post hoc test. Alterations of parameters between the first and second examinations in new‐onset T1D patients were compared based on paired *t*‐tests. Correlations were computed with Spearman rank order test. *P* values < 0.05 were considered statistically significant. Data are presented as means ± SEM.

### 2.7 Data and Resource Availability

The dataset and the resource used and/or analysed during the current study are available from the corresponding authors on reasonable request.

## 3 Results

### 3.1 Patient Population

In total, *n* = 6 HC, *n* = 4 new‐onset T1D (< 12 weeks from diagnosis) and *n* = 6 long‐standing T1D (> 10 years since diagnosis) patients were included. Among the new‐onset T1D participants *n* = 1 had hypothyroid disease (adequately substituted) and *n* = 1 epilepsy (well controlled on Fenfluramine). Among the participants with long‐standing T1D *n* = 3 had mild retinopathy. One of the healthy controls was treated with low‐dose Citalopram and one of the long‐standing T1D patients with low‐dose Escitalopram and Mirtazapine. As expected, HbA1c levels were higher and C‐peptide values lower in the T1D groups when compared to HC. Descriptive and metabolic data of all study participants is presented in Table 1.

**TABLE 1:** Descriptive data of study participants.

Parameter	Healthy controls (*n* = 6)	Long‐standing T1D (*n* = 6)	New‐onset T1D (*n* = 4)
Age (years)	28.2 ± 1.4	29.5 ± 1.6	34 ± 1.2*
Male gender (*n*, %)	4 (67%)	4 (67%)	3 (75%)
BMI (kg/m^2^)	25.7 ± 1.6	25.4 ± 1.3	22.6 ± 0.7
Disease duration (months)	N/A	240 ± 39	2 ± 0###
Creatinine (μmol/L)	82 ± 4	77 ± 2	70 ± 7
Haemoglobin (g/L)	135 ± 5	140 ± 3	137 ± 2
Leukocytes (× 10^9^/L)	5.4 ± 1.0	5.6 ± 0.3	3.8 ± 0.2
Platelets (× 10^9^/L)	261 ± 30	259 ± 22	206 ± 35
Neutrophils (× 10^9^/L)	3.1 ± 0.7	2.8 ± 0.3	1.9 ± 0.2
Eosinophils (× 10^9^/L)	0.11 ± 0.02	0.23 ± 0.05	0.08 ± 0.03^#^
Lymphocytes (× 10^9^/L)	1.8 ± 0.3	2.1 ± 0.1	1.4 ± 0.05
Monocytes (× 10^9^/L)	0.4 ± 0.04	0.5 ± 0.05	0.3 ± 0.04
F‐Glucose (mmol/L)	5.4 ± 0.1	7.7 ± 1.1	6.5 ± 0.7
HbA1c (mmol/mol)	33 ± 1	50 ± 2***	61 ± 4***^,^ #
F‐C‐peptide (nmol/L)	0.62 ± 0.08	0.01 ± 0.01***	0.28 ± 0.05***^,^ ##
AUC C‐peptide (nmol/L/2 h)	169 ± 12	2 ± 1***	78 ± 11***, ###
GADA positive (*n*, %)	0 (0%)	2 (33%)	4 (100%)
IA‐2 positive (*n*, %)	0 (0%)	5 (83%)	0 (0%)
ZnT8 positive (*n*, %)	0 (0%)	3 (50%)	3 (75%)

*Note:* For autoantibodies the determined detection limit from the Uppsala University Hospital clinical laboratory was applied; GADA > 5 IE/mL, IA‐2 > 7.5 kE/L and ZnT8 > 15 kE/L. C‐peptide was only detectable in *n* = 1 (detected value 0.02 nmol/L, detection limit 0.01 nmol/L) of the patients with long‐standing T1D. For the purpose of statistical comparison patients without detectable C‐peptide were assigned a numeric value of 0.01 nmol/L. The presented values for new‐onset T1D patients are from their first examination. A one‐way ANOVA with Tukeys's post hoc test was applied for statistical comparisons between the groups. Values are presented as mean ± SEM. * depicts *p* < 0.05, ** *p* < 0.01 and *** *p* < 0.001 when compared to HC and # depicts *p* < 0.05, ## *p* < 0.01, ### *p* < 0.001 when compared to long‐standing T1D patients.

### 3.2 Pancreatic [ ^68^Ga]‐DOTATATE and [ ^11^C]‐NES Uptake

**FIGURE 1:**
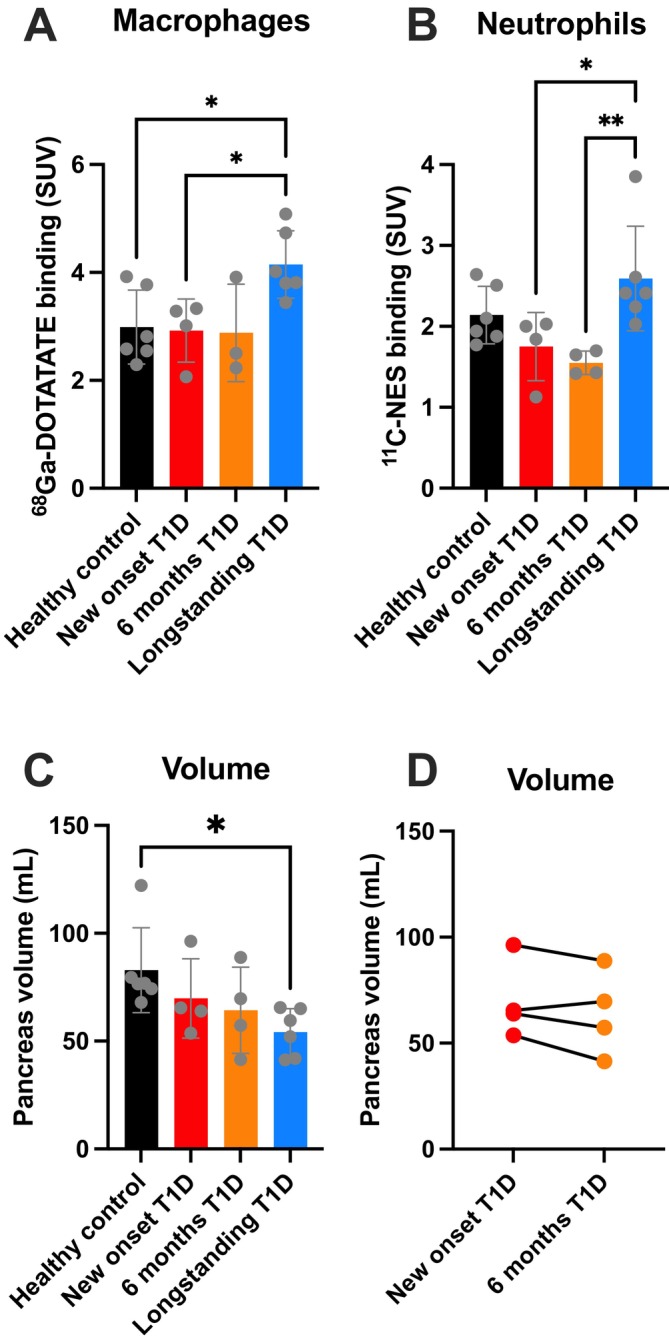
Standardised uptake values (SUV) of the M1 macrophage ^68^Ga‐DOTATATE and neutrophil elastase ^11^C‐NES PET‐tracers as well as the pancreas volume. Immune infiltration of M1 macrophages and neutrophils was examined in both healthy controls (*n* = 6), patients with new‐onset (*n* = 4) and long‐standing (*n* = 6) type 1 diabetes (T1D). Interestingly, we found that the pancreatic uptake was increased in patients with long‐standing T1D for both the M1 macrophage tracer (^68^Ga‐DOTATATE) (A) and the neutrophil elastase tracer (^11^C‐NES) (B) when compared to patients with healthy controls (HC) (macrophages only) and/or new‐onset T1D. There was, however, no difference in pancreatic uptake when comparing patients with new‐onset T1D to the healthy controls, and when re‐examined after 6 months, the uptake was similar in the patients with new‐onset T1D (due to a technical failure during the tracer synthesis, one of the patients with new‐onset T1D was only examined with ^11^C‐NES at the second examination). As expected, the pancreas volume was reduced in patients with long‐standing T1D when compared to healthy controls (C). In patients with new‐onset T1D, three out of four patients displayed a reduction of pancreas volume when they were re‐examined after 6 months, but there was no statistical difference in pancreas volume (probably due to the small sample size) (D).

We found both the pancreatic uptake of [^68^Ga]‐DOTATATE (Figure 1A) and [^11^C]‐NES (expressed as SUV_mean_, Figure 1B) to be increased in patients with long‐standing T1D when compared to patients with new‐onset T1D. For [^68^Ga]‐DOTATATE there was also an increase in binding in long‐standing T1D compared to HC. For [^11^C]‐NES there was no statistical difference compared to HC, but this could be reflected by the low number of study participants. In the new‐onset group there was no difference when comparing the first‐ and second examination within subjects.

### 3.3 Pancreas Morphology

In accordance with our own and previous reports, the pancreas in patients with long‐standing T1D was found to be smaller when compared to HC (Figure 1C). There was also a tendency towards a reduction of pancreas volume in new‐onset T1D patients when compared to HC but not statistically significant. In line with our previous findings a reduction in volume could be observed for three out of four patients with new‐onset T1D when re‐examined after 6 months (Figure 1D).

To evaluate whether the higher pancreatic SUV_mean_ in long‐standing T1D could be explained by reduced pancreas size, the total uptake (SUV_tot_) of each tracer was estimated by multiplying the SUV_mean_ with the pancreas volume (in mL). Despite the increased SUV_mean_ in long‐standing T1D, SUV_tot_ did not show a corresponding increase. For [^68^Ga]‐DOTATATE, SUV_tot_ was 253.7 ± 111.2 (*n* = 6) in HC, 209.4 ± 84.2 (*n* = 4) in new‐onset, and 221.1 ± 34.8 (*n* = 6) in long‐standing T1D. For [^11^C]‐NES, SUV_tot_ was 180.7 ± 67.7 (*n* = 6) in HC, 124.1 ± 52.6 (*n* = 4) in new‐onset, and 140.5 ± 42.4 (*n* = 6) in long‐standing T1D.

### 3.4 [ ^68^Ga]‐DOTATATE and [ ^11^C]‐NES Distribution in Other Tissues

**FIGURE 2:**
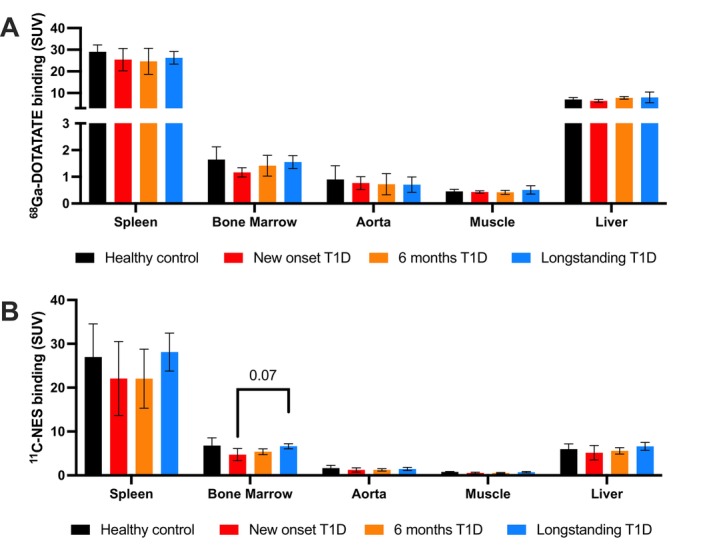
Standardised uptake values (SUV) of the M1 macrophage ^68^Ga‐DOTATATE and neutrophil elastase ^11^C‐NES PET‐tracers in major organs/tissues. There was no difference in the uptake of the M1 macrophage ^68^Ga‐DOTATATE PET‐tracer in any of the other reference tissues examined (A). For the neutrophil elastase tracer ^11^C‐NES, a greater variability was observed in the spleen, but there was no statistical difference between the groups (B). There was a tendency towards a slight reduction in the uptake in bone marrow in patients with new‐onset T1D when compared to patients with long‐standing T1D.

The uptake and signal were overall coherent in all examined tissues and for [^68^Ga]‐DOTATATE no differences were observed between the groups (Figure 2A). For [^11^C]‐NES the variation was greater in the spleen as compared to [^68^Ga]‐DOTATATE, but no significant differences were observed between the groups (Figure 2B). There was, however, a tendency towards a slightly lower uptake in the bone marrow of newly diagnosed T1D patients when compared to long‐standing T1D patients.

### 3.5 Correlations Between Imaging Assessments and Metabolic Markers

We found no correlation between markers of metabolic control and uptake of [^68^Ga]‐DOTATATE or [^11^C]‐NES when computing correlations for all study participants nor when computing correlations for patients with long‐standing T1D alone (data not shown). For [^68^Ga]‐DOTATATE a weak positive correlation was observed with the circulating levels of leukocytes (*r* = 0.49, *p* = 0.03), neutrophils (*r* = 0.47, *p* = 0.04), and eosinophils (*r* = 0.47, *p* = 0.043) when computing correlations for all study participants but not for long‐standing T1D alone. For [^11^C]‐NES no correlation was observed with the neutrophil count or any other of the analysed blood cells when computed for all participants or long‐standing T1D alone.

## 4 Discussion

In the current study we show that it is feasible to use PET/MRI and the PET‐tracers [^68^Ga]‐DOTATATE and [^11^C]‐NES to target pro‐inflammatory M1 macrophages and neutrophils, respectively, in the pancreas. However, we did not find an increase of neutrophils within the pancreas in patients with new‐onset T1D. In fact, we instead found that the uptake was higher for both [^11^C]‐NES and [^68^Ga]‐DOTATATE in patients with long‐standing T1D when compared to patients with new‐onset T1D. [^68^Ga]‐DOTATATE also demonstrated elevated pancreatic uptake in long‐standing T1D compared to HC. In an in‐depth characterisation of immune cells in pancreas biopsies from the Network for Pancreatic Organ Donors with Diabetes (nPOD), macrophages were more frequently identified in the pancreas of T1D patients compared to non‐diabetic controls, but the cell density was in fact higher in T1D patients with a longer duration [4], which is in line with our findings.

The number of circulating neutrophils has previously been found to be reduced in both pre‐symptomatic and new‐onset T1D, while the number of neutrophils within the pancreas seem to be increased in new‐onset T1D, based on data from pancreatic sections from brain‐dead organ donors [21]. It should, however, be noted that in the immunohistochemical studies of pancreas sections, the number of neutrophils was on average approximately three cells per mm^2^ [21], that is, although it represents an increase in numbers the total numbers were still small. Also, out of the five biopsies included from the nPOD and Siena cohorts that were considered as ‘long‐standing’ T1D, two of the donors had a disease duration of 1 year [21]. We designed our study so that all patients with long‐standing T1D had a disease duration > 10 years in order to exclude patients with a significant number of remaining beta‐cells. However, this introduces also the possibility of fibrosis development within the pancreas, which has been described to occur in long‐standing T1D and could perhaps explain the increase in both macrophages and neutrophils, since both of these cell types are known to play a role in end‐stage inflammatory processes and fibrosis [22, 23]. Importantly, macrophage polarisation in vivo is increasingly recognised as a continuum rather than a strict M1/M2 dichotomy [24]. Chronic fibrotic tissues are not immunologically inert but characterised by ongoing low‐grade inflammatory remodelling with heterogeneous macrophage populations that may retain expression of inflammatory receptors. Consistent with this concept, SSTR2‐targeted PET imaging with [^68^Ga]‐DOTATATE has been successfully used to detect macrophage‐associated inflammation in atherosclerotic plaques, which represent chronic inflammatory remodelling rather than acute inflammation [5]. Therefore, the increased pancreatic uptake observed in long‐standing T1D may reflect persistent inflammatory remodelling associated with pancreatic atrophy and fibrosis rather than acute insulitis.

**FIGURE 3:**
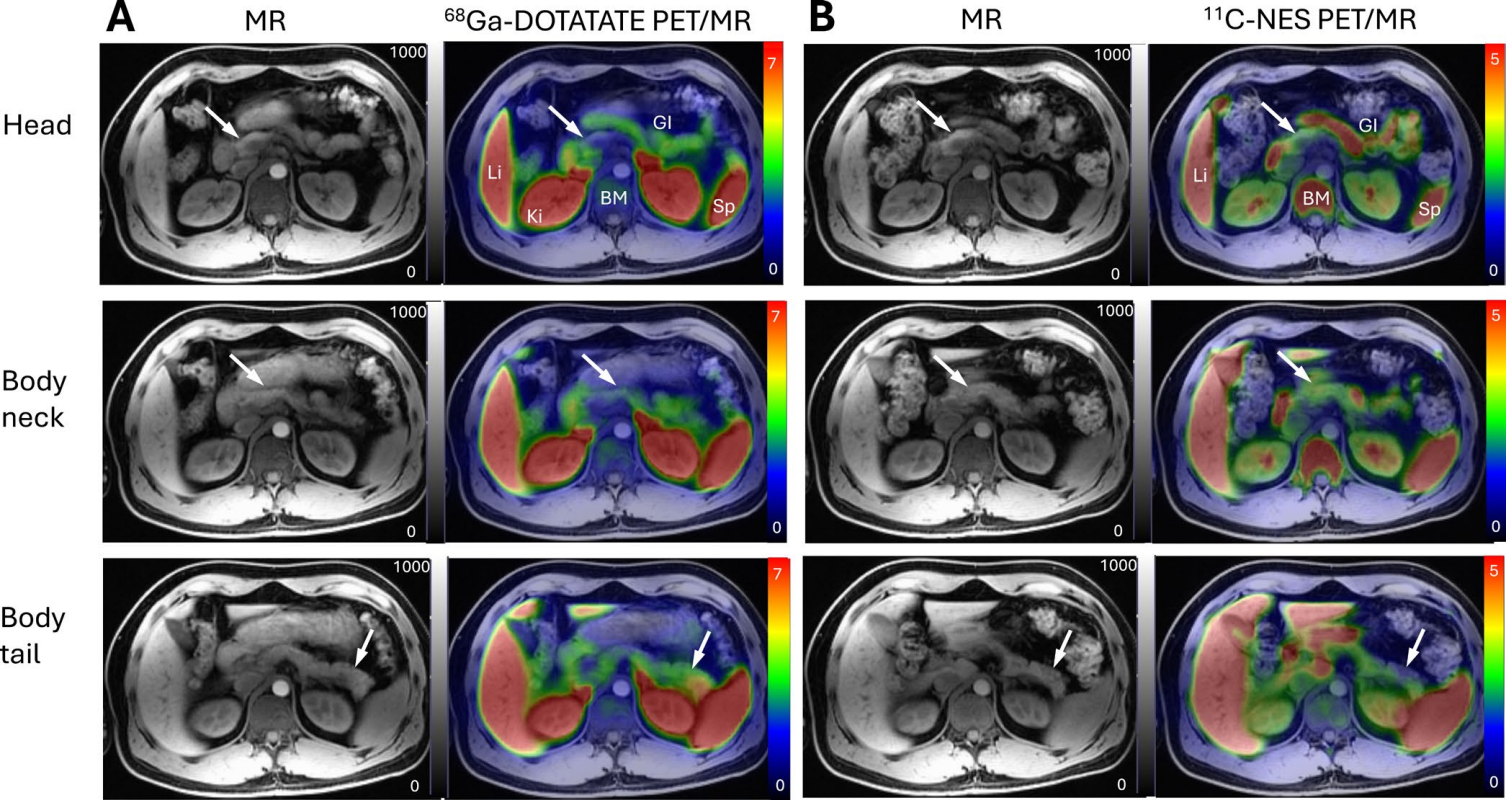
Representative PET/MRI images over the abdomen for the ^68^Ga‐DOTATATE and ^11^C‐NES PET‐tracers in a patient with long‐standing T1D. Both M1 macrophage marker ^68^Ga‐DOTATATE (A) and neutrophil marker ^11^C‐NES (B) demonstrated expected biodistribution and uptake in major abdominal organs. In pancreas, the binding of both ^68^Ga‐DOTATATE and ^11^C‐NES visually exhibited patchiness with some regions showing higher uptake and some regions having virtually no uptake. White arrows indicate pancreas on transaxial projections at the level of the pancreas head (caput), body (corpus) and tail (cauda).

Previous studies based on technetium‐labelled human polyclonal immunoglobulins have shown that there is a clear accumulation of signal in seven out of 15 patients with new‐onset T1D [25]. Interestingly this signal persisted 1 year after diagnosis but was associated with a higher frequency of partial clinical remission. The accumulation of immunoglobulins was not correlated with titers of autoantibodies. It was speculated that the accumulation of immunoglobulins is mediated by non‐specific factor such as an increased vascular permeability due to inflammation. Also, in a clinical study based on MRI and the uptake of the magnetic nanoparticle ferumoxytol which accumulates in macrophages an increased pancreatic signal in patients with new‐onset T1D was observed [26]. Interestingly, in this study a clear heterogeneity both in‐between and within subjects was identified. Out of the *n* = 11 new‐onset T1D patients, two had a distinct increase of signal which was however heterogeneously distributed within the pancreas. Also, when reviewing the images presented in the article by Baron et al. [25] it is clear that the uptake within the pancreas is heterogenous. Although we did not observe an increased uptake of either [^68^Ga]‐DOTATATE and [^11^C]‐NES in patients with new‐onset T1D we do find an increased signal in patients with long‐standing T1D and when reviewing the image material an heterogenous uptake within the pancreas does appear (Figure 3A,B). However, we did not perform a strict head–body–tail subdivision of the pancreatic VOIs. This decision was driven primarily by the risk of spill‐in/partial volume effects from adjacent high‐uptake structures, most notably the spleen and small bowel. To maximise robustness and inter‐subject comparability, we applied a conservative segmentation strategy and delineated pancreatic regions where anatomical borders were clearly defined on MRI and where signal contamination from neighbouring organs was negligible. Given the limited sample size and the expected effect size, a segment‐level (body vs. tail) analysis would likely have had insufficient statistical power and would substantially increase measurement variability and multiple‐comparison burden. Therefore, segmental analyses were not included as exploratory outcomes.

Considering that the applied imaging protocol is non‐invasive and minimises the use of ionising radiation, it could also be applied for repeated studies of patients with pre‐symptomatic T1D. Since we have proved that it is feasible to study immune infiltration of the pancreas, it also opens up for the use of additional tracers to study the dynamics of the adaptive immune system, of which it might be most interesting to target CD8+ T‐cells. This has recently been demonstrated to be feasible in a clinical setting even for whole‐body visualisation of CD8+ T‐cells by the use of a zirconium‐89‐labelled CD8‐specific one‐armed antibody tracer (^89^ZED88082A) [27]. The data presented holds promise for validating this tracer for targeting CD8+ T‐cells also for studies of immune infiltration within the pancreas during T1D progression. However, the higher radioactive dose of long‐lived PET radionuclide Zirconium‐89 may restrict repeated scanning depending on the study protocol.

In conclusion, we demonstrate the feasibility of non‐invasive in vivo visualisation of M1 macrophages and neutrophil‐mediated inflammation within the pancreas in T1D. However, our findings do not support an increase in said immune cell infiltration or activity within the pancreas of adult patients with new‐onset T1D. Instead, we found an increase in patients with long‐standing T1D, which could be representative of an end‐stage inflammatory process and fibrosis.

## Author Contributions

D.E. O.K. and O.E. conceptualised and designed the study. H.H. was responsible for patient inclusion and follow‐up. I.V. was responsible for radiolabeling development. H.Z., P.L. and N.G.M. analysed MRI data. H.Z. and O.E. analysed the PET‐image data. E.A. collected and analysed clinical data. O.E., E.A., H.Z. and D.E. analysed the combined data‐set and interpreted the data. E.A., H.Z., O.E. and D.E. drafted and wrote the manuscript. All authors critically reviewed and edited the manuscript and approved of it prior to submission. D.E. is the guarantor of this work and, as such, had full access to all the data in the study and takes responsibility for the integrity of the data and the accuracy of the data analysis.

## Funding

The study was funded by Barndiabetesfonden, Uppsala Diabetes Center, Diabetesfonden, Science for Life Laboratory, the Swedish Research Council (Vetenskapsrådet) (2020–0231 and 2024–03659; OE, 2019–01415 and 2023–02221; OK), Swedish Cancer Society/Cancerfonden (24 3754 Pj; OE and Cancerfonden 21 1519 Pj; OK), an EFSD/Lilly grant, ExoDiab, the Ernfors Family Fund, and Göran Gustafssons Stiftelse.

## Disclosure

Olof Eriksson is a part‐time employee of Antaros Tracer AB. Otherwise, the authors have nothing of relevance to this study to disclose.

## Conflicts of Interest

O.E. is an employee of Antaros Tracer AB. The other authors declare that they have no competing interests.

## Data Availability

The dataset and the resource used and/or analyzed during the current study are available from the corresponding authors on reasonable request.
